# Long-term outcome following upper extremity replantation after major traumatic amputation

**DOI:** 10.1186/s12891-017-1442-3

**Published:** 2017-02-10

**Authors:** G. Mattiassich, F. Rittenschober, L. Dorninger, J. Rois, R. Mittermayr, R. Ortmaier, M. Ponschab, K. Katzensteiner, L. Larcher

**Affiliations:** 1 Trauma Center, Unfallkrankenhaus Linz-Teaching Hospital of the Paracelsus Medical University Salzburg, Garnisonstrasse 7, 4010 Linz, Austria; 2 Ludwig-Boltzmann Institute for Experimental and Clinical Traumatology, Vienna, Austria; 3 Department of Orthopaedic Surgery, Ordensklinikum Linz Barmherzige Schwestern - Teaching hospital of the Paracelcus Medical University Salzburg, Linz, Austria; 4 Trauma Center Vienna Meidling, Vienna, Austria; 5 Department of Trauma Surgery, Paracelsus Medical University and Salzburger Landeskliniken, Salzburg, Austria; 6 Trauma Center Salzburg, Teaching Hospital of the Paracelsus Medical University Salzburg, Salzburg, Austria; 7 Plastic, Aesthetic and Reconstructive Surgery Sanitaetsbetrieb South Tyrol (SABES), Bolzano, Italy

**Keywords:** Amputation, Macro-amputation, Replantation, Macro-replantation, Long-term results, Upper extremity, Microsurgery

## Abstract

**Background:**

Amputations in general and amputations of upper extremities, in particular, have a major impact on patients’ lives. There are only a few long-term follow-up reports of patients after macro-replantation. We present our findings in contrast with the existing literature.

**Methods:**

Sixteen patients with traumatic macro-amputation of an upper extremity were eligible for inclusion in this study. Altogether, the patients underwent replantation in 3 institutions between 1983 and 2011.

**Results:**

Twelve male and four female patients with an average age at injury of 40.6 years (range, 14–61 years) were included in this study. The mean follow-up period was 13.5 years (range, 4.4–32.6 years; SD, 5.7 years). The mean disabilities of the arm, shoulder and hand (DASH) outcome measure was 41 (range, 5.2–94.8; SD, 18.2), functional independence measurement (FIM) was 125 (range, 120–126; SD, 1.8). Chen I representing very good function was accounted in six, Chen II representing good function in eight, Chen III (fair) in one and Chen IV (bad function) in one patient.

**Conclusions:**

We found that while the majority of the included patients exhibited good or very good function of the extremity, none of the replanted appendages regained normal levels of functionality. In addition, all participants were very satisfied with their outcomes. Positive long-term results with high rates of subjective satisfaction are possible after replantation of upper extremities.

## Background

Amputations in general and amputations of upper extremities, in particular, have a major impact on patients’ lives, as loss of function can not only cause reduced autonomy in daily life but also hinder social interactions and capacity for work.

Although the numbers of major traumatic amputations have been declining over the years as a result of continuous progress in occupational safety activities, major amputations of upper extremities are reported to have an average prevalence of 11.6/100.000 individuals in Europe [1]. Young, active males are more often affected by upper extremity amputation, which often result from high-energy trauma.

As major amputations are often accompanied by multiple, life-threatening injuries following high-energy trauma, the possibility for replantation in these patients is restricted to prevent further harm caused by additional systemic problems occurring after revascularization.

Successful replantation after major upper extremity amputation is possible in 77–93% of cases [2–6].

Although modern prosthetic devices have improved over recent years, high rejection rates are still observed in patients supplied with prostheses; thus, replantation of the lost extremity is still believed to yield better overall subjective results [7].

There are only a few long-term follow-up reports of patients after macro-replantation. The intention of this study was to present long-term results after macro-amputation of an upper extremity. We present our findings in contrast with the existing literature. These results may help to determine factors that could influence a surgeon’s decision regarding management.

## Methods

### Patients

Twenty appropriate patients were identified in the archives of three different trauma centers of the Austrian Social Insurance for Occupational Risks (AUVA).

Sixteen patients with traumatic macro-amputations were eligible for inclusion in this study. Altogether, the patients underwent replantation of an upper extremity in 3 institutions of the AUVA between 1983 and 2011. One patient was excluded due to a need for secondary amputation. Two patients died from malignoma unrelated to their injury, and one patient refused to participate in the study for unknown reasons. Twelve male and four female patients with an average age at injury of 40.6 years (range, 14–61 years) were included in this study. The mean follow-up period was 13.5 years (range, 4.4–32.6 years; SD, 5.7 years).

All patients were psychologically stable and did not suffer from serious systemic disabilities at the time of injury; therefore, they were all suitable candidates for replantation and subsequent reconstructive procedures. Moreover, these patients showed good compliance during the post-operative phase and rehabilitation.

All operation reports and post-operative documents were analyzed with respect to the mechanism of injury, time between injury and operation, duration of replantation procedure, extent of soft tissue damage, accompanying injuries, post-operative complications and secondary procedures.

### Scores and classifications

The functional outcomes were assessed using the disabilities of the arm, shoulder and hand (DASH) outcome measure [8], the functional independence measurement (FIM) [9], and clinical examination. Extremity function was graded according to the system described by Chen. [10] A structured interview was performed with all patients, focusing on the evaluation of subjective satisfaction and the ability to perform daily activities.

### Informed consent

All patients provided written, informed consent after being informed about the protocol and purpose of the study. It was approved by the ethics Committee of the Austrian social insurance for occupational risks (AUVA) that personal rights were respected in this study.

## Results

All patients were operated on between 1983 and 2011 at our institutions. The time between the trauma and the beginning of the operation ranged from 38 min to 3 h and 50 min (average, 1.58 h; SD, 35 min). The operation duration ranged between 1:42 h and 13:35 h (average, 7:32 h; SD, 2:31). The length of hospital stay was on average, 40 days (SD, 18.5 days). Subtotal amputation was found in 5 cases, with remaining skin bridges ranging from 5–15 cm in length. The region of amputation was the upper arm in 8 cases, the forearm in 5 cases, and the wrist in 3 cases. Relevant accompanying injuries were found in 2 patients; one patient suffered from hemorrhagic shock, and one patient suffered from a pneumothorax in need of drainage. The cause of injury was an industrial machine in 6 cases and a wood splitter or circular saw in 7 cases. Two other patients were each involved in a car accident, and one patient was trapped by stones. (Tables 1 and 2)

**Table 1:** Epidemiological data

Pat. no.	Gender	Injury pattern	Age at accident	Handedness	Affected side	Follow-up period
1	F	Avulsion	50	R	L	7.5
2	M	Sharp cut	36	R	L	16.5
3	M	Sharp cut	47	R	R	4.4
4	M	Crushed	44	R	L	17.3
5	M	Avulsion	21	R	R	12.3
6	M	Avulsion	56	R	L	12
7	M	Sharp cut	36	R	L	9.3
8	M	Sharp cut	61	R	R	15.3
9	M	Avulsion	57	R	R	15.8
10	F	Sharp cut	14	R	L	32.6
11	F	Crushed	14	R	L	6.7
12	M	Sharp cut	53	R	R	6.5
13	f	Sharp cut	37	R	R	24.3
14	M	Sharp cut	54	R	R	5.3
15	M	Sharp cut	38	R	L	12.5
16	M	Avulsion	31	R	R	8.3

*Pat. No.* Patient number, *M* male, *F* female, age at accident presented in years, *R* right side, *L* left side, Follow-up period presented in years

**Table 2:** Overview of injury pattern, duration of operation and secondary reconstruction procedures

Pat. no.	Amputation level	Extent of amputation	Time to operation theatre	Duration of replantation	Secondary reconstruction
1	Upper arm	Skin bridge (ulnar nerve damaged, but in continuity)	02:30	08:30	Functional latissimus dorsi transfer + sural nerve grafting
2	Upper arm	Total	02:00	09:15	Functional latissimus dorsi transfer
3	Wrist	Total	02:16	13:35	Sauvé-Kapandji procedure
4	Upper arm	Skin bridge of 15 cm	03:00	10:00	Radial nerve replacement, functional latissimus dorsi transfer
5	Upper arm	Subtotal	00:38	07:30	Sternocleidomastoid flap, brachial plexus reconstruction
6	Upper arm	Subtotal	00:45	08:45	Skin grafting
7	Upper arm	Subtotal	01:00	03:18	Sural nerve grafting
8	Wrist	Total	02:00	10:50	Arthrodesis wrist joint
9	Forearm	Total	01:30	11:55	Functional latissimus dorsi transfer
10	Elbow	Total	02:10	05:15	Tendon transfer
11	Forearm	Subtotal	02:22	04:11	None
12	Forearm	Total	01:40	06:58	Gore-Tex vessel graft
13	Forearm	Total	03:50	06:40	Carpo-metacarpal joint arthrodesis
14	Distal upper arm	Total	02:01	05:24	Functional gracilis flap
15	Wrist	Total	02:10	01:42	Scaphoid screw fixation, tenolysis, extensor tendon reconstruction
16	Upper arm + forearm	Upper arm: skin bridge of 7 cm Forearm: subtotal	01:37	06:40	Rotation flap upper arm

### Clinical results

The mean DASH score was 41 (range, 5.2–94.8; SD 18.2), and the mean FIM was 125 (range, 120–126; SD, 1.8). Six patients exhibited very good function, represented by Chen I; eight patients exhibited good function, represented by Chen II; and the two remaining patients exhibited either fair or bad function, represented by Chen III and Chen IV, respectively. Pain on the visual analogue scale (VAS) was 1.2 at rest (range, 0–6; SD, 1.5) and 4.1 during motion (range, 0–10; SD, 2.3). Six patients had protective sensitivity, two reported sensitivity close to normal, and one patient exhibited parasthesia. Intolerance to coldness was declared by all patients. Additionally, 7 patients claimed meteorological sensitivity. Interestingly, all patients stated that they would undergo replantation again, regardless of their functional result.

All but one patient regained protective sensation. Table 3.

**Table 3:** Clinical examination results

Pat. no.	DASH	FIM	Chen	VAS at rest	VAS under exertion	Ability to grip forceps	Sensitivity	Meteoropathy	Would undergo surgery again
1	46.7	126	II	5	6	Y	Protective	Y	Y
2	14.2	126	I	2	2	N	Protective	Y	Y
3	61	126	I	1	1	Y	Normal	N	Y
4	60.8	122	II	0	5	Y	Protective	Y	Y
5	42.5	120	IV	2	10	N	None	Y	Y
6	53.3	121	II	3	6	Hardly	Allodynia, decreased thumb sensitivity, normal at rest	Y	Y
7	11.67	126	II	0	0	Y	Slightly decreased	Y	Y
8	48.33	126	II	0	3	Y	Normal	Y	Y
9	40	126	III	0	5	Y	Protective	Y	Y
10	28.4	126	I	0	2	N	Slightly decreased	Y	Y
11	19.2	126	I	0	0	N	Normal	N	Y
12	56	121	II	0	4	N	Slightly decreased	Y	Y
13	51.7	125	II	6	9	N	Sharply decreased	Y	Y
14	94.8	126	II	0	5	N	Decreased	Y	Y
15	5.2	126	I	0	5	Y	Normal	N	Y
16	22.5	126	I	0	3	Y	Decreased	Y	Y

*Y* yes, *N* no, *VAS* visual analog scale

### Case one

A 56-year-old male patient sustained a total amputation at the level of the right upper arm after a cable was wrapped around it in an occupational injury. The patient had complete disruption of the muscles, vessels and nervous structures, as well as a fracture of the humerus in the distal third.

The patient had no other injuries and was otherwise fit and healthy. Immediate preparation of the stump and the amputated arm was performed by two separate teams. After debridement, bone resection of the humerus and identification of the vascular and nervous structures was performed, and a stable osteosynthesis with a 4.5-mm plate was executed. Subsequently, an anastomosis of the brachial artery was completed. Successful revascularization was achieved 4.5 h post trauma. Revascularization was followed by anastomosis of the veins, immediately after which a veno-venous hemodiafiltration was initiated. Then, an epineural suture of the radial, ulnar and median nerves was performed, and the muscles were readapted. A fasciotomy of the lower arm and carpal tunnel release were also performed. The skin was left open. The procedure took 5.5 h. Post-operatively, the patient showed good re-capillarization of the fingers and physiotherapy was initiated to mobilize the fingers. On the 4th post-operative day, a skin graft was used to close the wound. The patient developed neuropathic pain of the ulnar and median nerves with a positive Hoffmann-Tinel sign, which was treated by opioid therapy. The patient was transferred to rehab and showed good progress in elbow movement; however, bending of the thumb and fingers was not possible, although intensive therapy had been performed. This was the indication for the functional transfer of the gracilis muscle with adaption to the deep antebrachial flexor tendons and nerval anastomosis to the ulnar nerve.

**Fig. 1:**
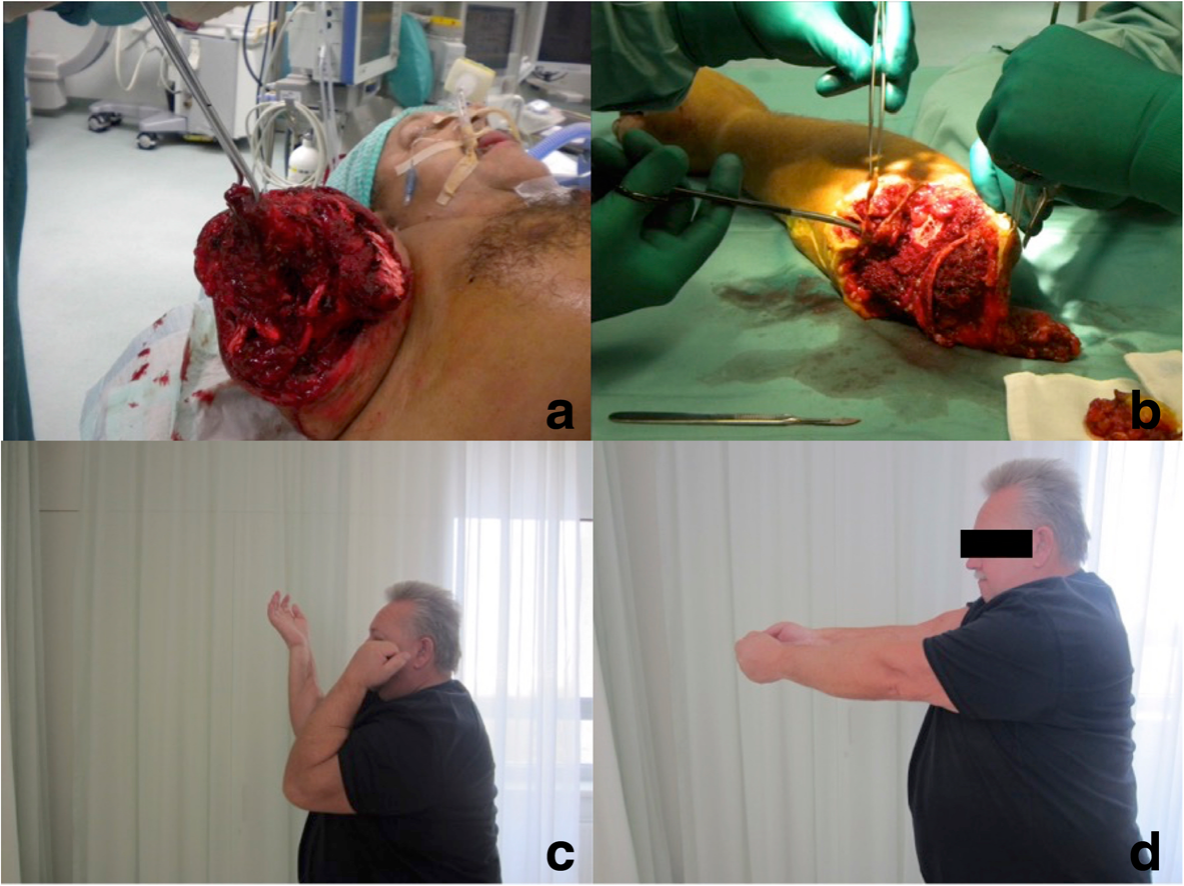
Case one – Total amputation of the upper arm. **a** preoperative stump. **b** preparation of the amputate. **c** flexion deficit of the elbow. **d** Full extension of the elbow

Six years after the trauma, the sensitivity of the fingers was almost normal, and prehensility had been preserved. Although the patient was unable to work after the injury, he was otherwise relatively satisfied and able to independently perform daily activities. He displayed a good capacity for shoulder and elbow motion. Wrist extension was possible, but both wrist flexion and finger bending were reduced. If needed, he would undergo replantation again (Fig. 1a–d).

### Case two

The lower arm of a 14-year-old female patient was trapped two stones; she sustained a subtotal amputation with a skin bridge of 3 cm, remaining tendon of extensor carpi radialis muscle, and complete ischemia of all fingers. An osteosynthesis was performed after debridement and shortening of the bone. Then, anastomosis of the radial and ulnar arteries was performed. Four and a half hours after the trauma, revascularization was successful. Subsequently, venous and nervous anastomosis was performed, muscles were readapted, and tendons were sutured. The skin was left open.

Post-operative recovery was uneventful, and physiotherapy and specialized hand therapy was initiated.

**Fig. 2:**
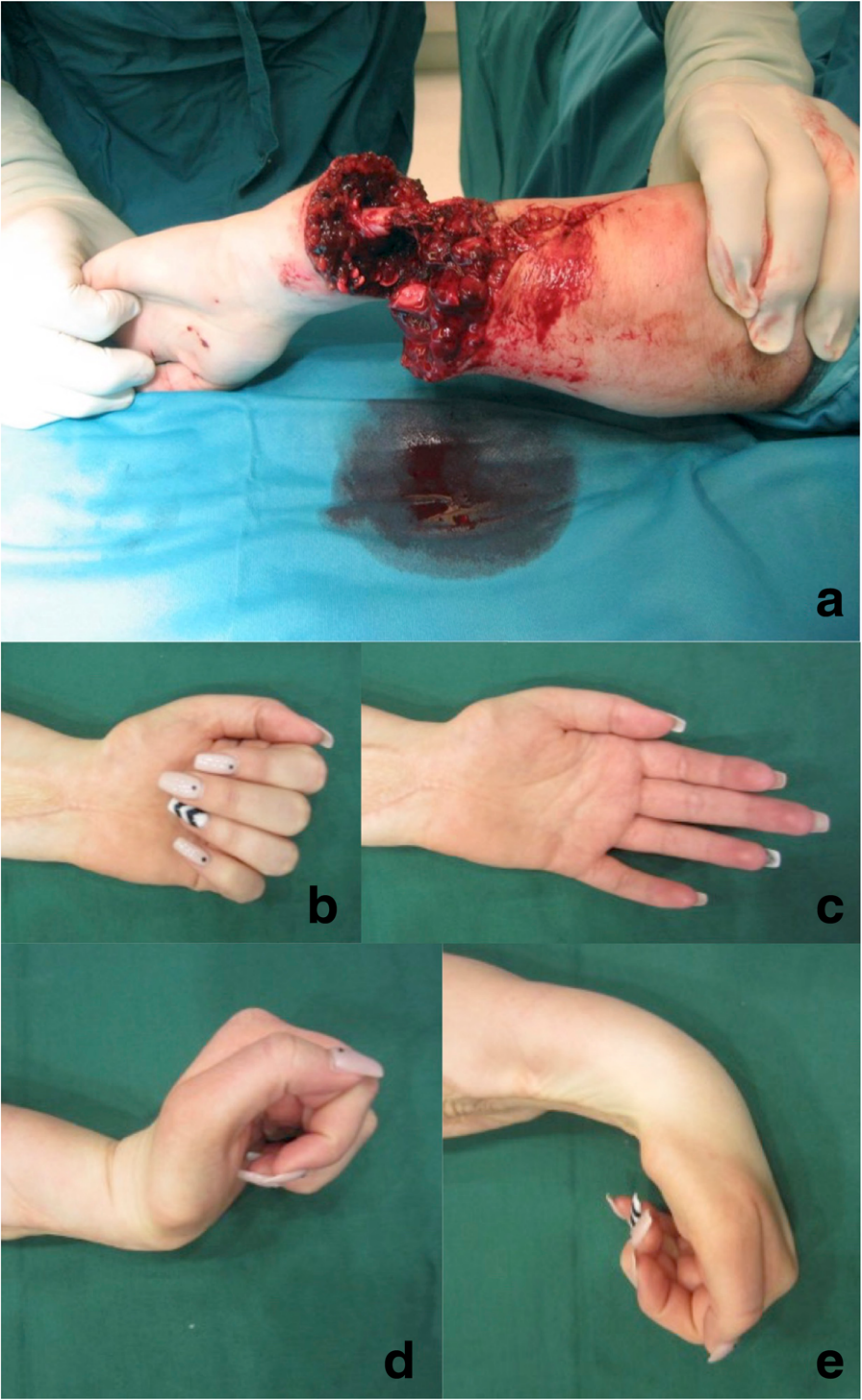
Case two – Subtotal amputation of the forearm. **a** preoperative picture with 3 cm skin bridge. **b** and **c** functional recovery with full range of motion of the fingers. **d** and **e** function recovery with slightly reduced flexion and extension of the wrist

Seven years after replantation, the flexion of all fingers was possible. The patient had normal finger sensitivity. Opposition of the thumb was reduced, but this did not subjectively affect the function of the hand. The patient is extremely satisfied and happy with the outcome (Fig. 2a–e).

### Case three

**Fig. 3:**
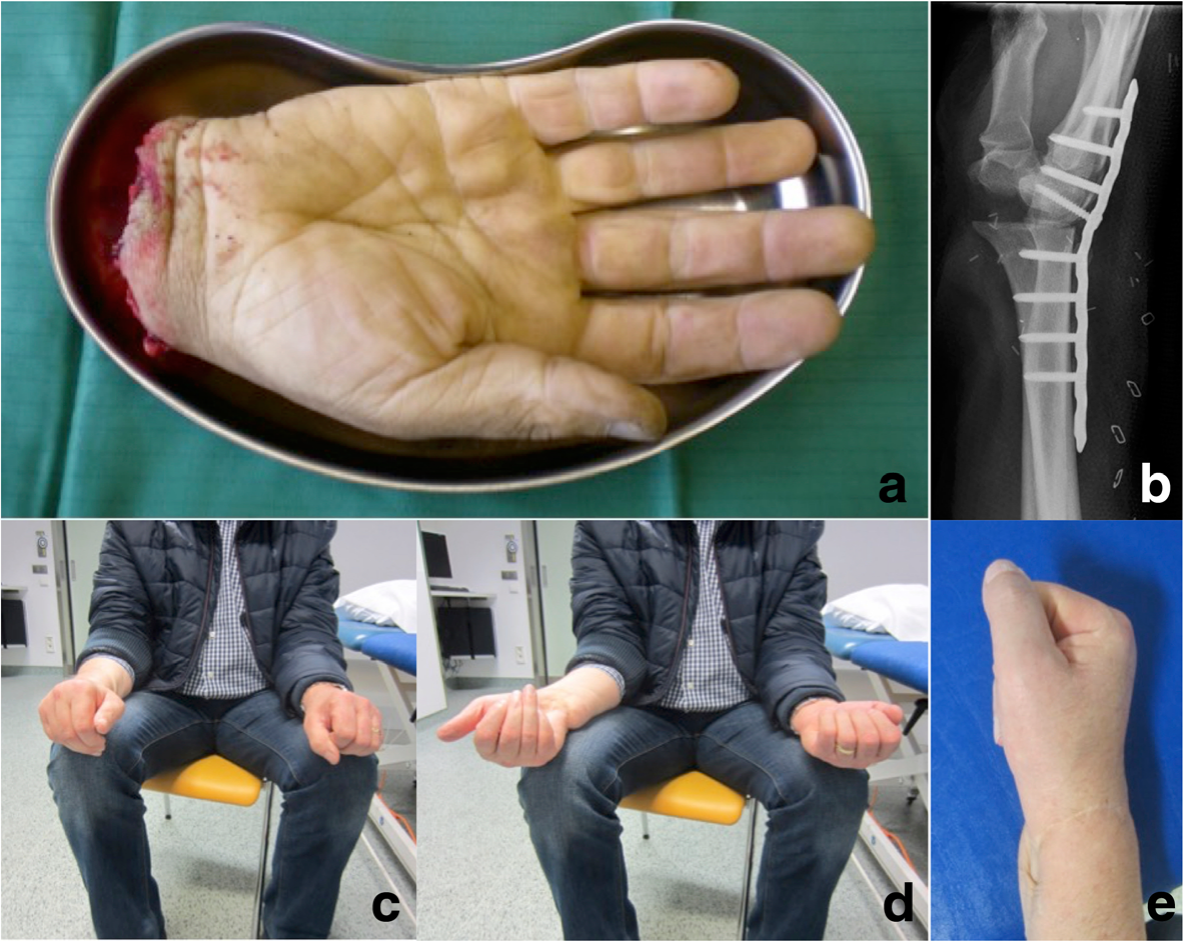
Case three – Total amputation at the level of the wrist joint. **a** preoperative picture of the amputate **b** post-operative X-ray after arthrodesis of the wrist-joint. **c** Slightly reduced flexion of the fingers and forearm pronation on the affected *right side*. **d** Full range of forearm supination. **e** fist closure in neutral position

A 47-year-old male patient underwent a work-related total amputation with a circular saw at the level of the wrist joint of the right hand. The patient was transported to the hospital and showed no other injuries. The otherwise healthy patient was then transferred to the operating theatre. The time from injury to operating theatre was 2:16 h. After debridement of the wound, a proximal row carpectomy and arthrodesis with a locking compression plate had to be performed. Microsurgical reconstruction of the ulnar and radial arteries and of the ulnar, median and radial nerves was performed. Then, all extensor and flexor tendons were sutured. The skin was left open on the volar side, and the hand was placed in a cast. The operation took 13:35 h. For the post-operative phase, the patient was transferred to the intermediate care unit and was given a catheter for axillary pain. On the third post-operative day, a skin graft was used to close the skin. Psychotherapy and physiotherapy were initiated on the fourth post-operative day. Intensive hand therapy was continued throughout the hospital stay. Wound healing was uneventful. The patient was discharged 21 days after the injury. Ambulatory rehabilitation was continued, and the patient showed good progress in reinnervation and motion of the fingers, but had restricted supination. Six months after replantation, resection of the ulnar head and tenolysis were performed. The patient returned to work as an office worker and farmer one year after sustaining the injury. The follow-up examination 53 months after replantation showed an excellent range of motion of the fingers (fingertip-palmar distance of under 1 cm), a sensitivity in all fingers close to normal (2-point distinction of 10 mm), and an almost normal rotation of the forearm compared to the contralateral side. The patient had a VAS rating of one and did not need analgesic medication. He appeared to be in good physical condition, competent and capable, and he declared his satisfaction with both the procedure and rehabilitation (Fig. 3a-e).

## Discussion

### Overall findings

The long-term, subjective functionality of replanted upper extremities is satisfying both for patients and surgeons, and functionality has not seemed to decrease over the years following the operation. The patients reported to have had improvements in function for up to two years post trauma. In addition, the patients appear to have adapted to impaired function, even if the objective utility was reduced. All of our examined patients would (if necessary and possible) undergo replantation again; post-operative recovery programs may involve the need for secondary reconstructive surgical procedures, and unexpected future complications are possible. None of our patients would opt for secondary amputation of the replanted extremity.

Articles on major limb replantation predominantly examine crushing or avulsion injuries; these studies report high levels of patient satisfaction and reasonable functionality of the replanted upper extremity [6, 11, 12].

### History and long-term results

The first report of replantation of an upper extremity was made by Malt in 1962; the patient was a young male [13]. Chen performed the first successful hand replantation in China in 1964 [14]. With advances in fixation devices, microsurgical techniques and reconstructive options, subjective and objective outcomes can be reasonable and are (to date) preferable to those of prostheses [15, 16]. Compared with amputations above the elbow, better results have been obtained after sharp and distal injuries [17]. Good functional recovery after replantation can only be achieved under optimal circumstances involving experienced surgeons. If hand prehensility is preserved, a superior functional outcome of the replanted upper extremity can be expected [2].

In terms of use of the extremity, the functional outcome mainly depends on restoration of the preserved musculo-tendinous units, tissue coverage and sensation.

Overall, reconstruction of an upper extremity seems beneficial [2, 16, 17]. The best outcomes can be achieved in more distal amputations and in children [18–20].

Hoang [21] reported the outcomes of five consecutive hand replantations at the level of the radio-carpal joint. These all resulted from clean-cut amputations in young Vietnamese males and were replanted within 9 to 14 h of the injury. With an average follow-up period of 33 months, the patients exhibited 70 to 80% total active motion of the digits and thumb opposition compared with that of the contralateral hand, as well as 8 to 12 mm of two-point static discrimination.

Blomgren et al. [22] reported success rates of 92% for incomplete and 71% for complete hand replantations at different levels of injury.

Amputations proximal to the elbow tend to have disappointing functional results [23]. Secondary reconstructive surgical procedures are often needed to improve function.

While isolated amputation of a hand or upper extremity causes tremendous problems concerning functionality and psychological distress, it is not a primarily life-threatening event [24].

Gulgonen and Ozer et al. [4] presented the long-term results of major upper extremity replantations of 9 patients 18 years after injury. They concluded that the replantation of an upper extremity proximal to the wrist joint satisfactorily restored upper extremity function. Functional recovery was better in replantations distal to the mid to distal forearm.

Daoutis et al. [6] reported functional results after replantation/revascularization in 47 cases using Chen’s criteria for evaluation. They included patients with amputations proximal to the metacarpophalangeal joints as well as incomplete amputations. Overall, 42 successful replantations were evaluated after an undisclosed time, and only 5 patients out of the 42 listed did not have use of their replanted limb.

In their evaluation of 17 successfully replanted extremities, Laing et al. demonstrated favorable or acceptable long-term results in the majority of cases after a mean time of 5.4 years. They concluded that high patient satisfaction rates emphasize the positive psychological impact of successful replantation [3]. Body image and self-awareness are also important factors for patient satisfaction.

### Decision making

As major amputations occur frequently with high-energy trauma, accompanied by various and occasionally life-threatening injuries, it is important to primarily address those injuries to save the patient’s life [25].

The decision between salvaging or amputating a limb must be made rapidly. Although therapeutic algorithms have been proposed in the lower as well as in the upper extremity, [26–28] this decision has to be made on a case-by-case basis and has been shown to not be supported by scoring systems, as it is in the lower extremity [29, 30]. In addition, the number of factors influencing the function of the upper compared with the lower extremity has to be considered.

Advanced, interdisciplinary surgical skills are required in these cases, as well as specialized infrastructure and equipment.

### Secondary reconstructions

After a successful upper extremity replantation, patients often need secondary reconstructive interventions for soft tissue coverage, functioning muscle transfers, tendon transposition or tenolysis. Fufa et al. [31] evaluated secondary reconstructive interventions in 40 patients after successful replantation and found that the average number of secondary surgical procedures was three per patient. They proposed a treatment algorithm for the management of major upper limb replantation. They suggested that the type of reconstructive surgery could be based on the pattern of injury, which depends on the zone and level of the injury.

### Improvements in post-operative intensive care

In addition to improvements concerning surgical techniques, intensive care management has also been enhanced.

Due to several systemic metabolic changes and the release of oxidized free radicals, patients might develop systemic reperfusion injuries following surgery [15].

Replantation of an upper limb also bears the risk of local or systemic complications, such as sepsis, rhabdomyolysis with renal failure or delayed wound healing.

Post-operative management has to be interdisciplinary, and it is of the utmost importance to consider the general condition of macro-amputation patients, while also avoiding collateral harm to the patient when saving the limb.

After replantation, all patients in this study began therapy under the supervision of a specialized hand therapist.

### Prosthetic technology

Improvements in prosthetic technology have been made in the past decade. Especially sensory feedback of the hand is still only partially replicated by the newest prosthetic technology. This might be the reason why upper limb prostheses have high rejection rates of more than 30% [32, 33].

Prehensile function and the sensation of touch are technically difficult to regain [34].

The prosthesis acceptance rates illustrate the psychological willingness of patients to function single-handedly, rather than to use a burdensome, non-intuitive prosthetic limb [34].

Cost-benefit analyses of performing replantation vs. shortening and closing the amputation stump, patient satisfaction levels, and the incidence of post-replantation problems (e.g., cold tolerance) have been studied in different countries and health care systems, with varied results. Further developments of prosthetic devices with targeted muscle reinnervation will have a positive impact on the management of amputations, and prostheses remain good alternatives if replantation is not possible.

### Limitations and strengths of the study

This study is naturally limited by the relatively small and heterogeneous group of patients. However, every replantation patient has had a relatively unique pattern of injury. All but one patient wanted to participate in the study, and all were highly motivated to share their experiences. We were able to review the documentation electronically and can therefore guarantee the thoroughness of the documentation.

## Conclusions

We found that while the majority of the included patients exhibited good or very good function of the extremity, none of the replanted appendages regained normal levels of functionality. All participants were very satisfied with their outcomes.

Although 39–79% of all replantation patients suffer from pain, only a few patients want to undergo a secondary amputation of the replanted extremity [34]. As validly stated by Sterling Bunnell, a “bad hand” is functionally better than a “good amputation.”

Due to the infrequency of these replantations, they should be performed at specialized replantation centers to concentrate the expertise and to achieve the best possible outcomes.

Our patients were very satisfied with their replanted upper limbs, which have helped them regain a quality of life better than what they might have otherwise had.

A positive long-term result with a high rate of subjective satisfaction is possible after the replantation of an upper extremity.

Developments in microsurgical techniques and devices may positively affect the results and development of prosthetic devices. Nearly natural movements, greater prehensility and artificial (but practical) functionality may also have an impact on the treatment of such patients.

In this comparatively small but varied collection of case studies, there was no significant influence found on the functional results regarding (i) level of amputation, (ii) time of ischemia or (iii) age of the patient.

Major limb replantation can yield favorable long-term functional results that together with psychological benefits, make it a feasible option in selected patients.

## Abbreviations

AUVA: Austrian social insurance for occupational risks
DASH: Disabilities of the arm, shoulder and hand
FIM: Functional independence measurement
VAS: visual analogue scale
